# The Retention Effect of Resin-Based Desensitizing Agents on Hypersensitivity—A Randomized Controlled Trial

**DOI:** 10.3390/ma15155172

**Published:** 2022-07-26

**Authors:** Manami Tadano, Tomoaki Nakamura, Seira Hoshikawa, Ryoko Hino, Yuriko Maruya, Aya Yamada, Satoshi Fukumoto, Kan Saito

**Affiliations:** 1 Division of Pediatric Dentistry, Department of Oral Health and Development Sciences, Tohoku University Graduate School of Dentistry, Sendai 980-8575, Japan; manami.tadano.e5@tohoku.ac.jp (M.T.); tomoaki.nakamura.d2@tohoku.ac.jp (T.N.); seira.hoshikawa.e5@tohoku.ac.jp (S.H.); ryoko.m@dent.tohoku.ac.jp (R.H.); yuriko.maruya.c1@tohoku.ac.jp (Y.M.); yamada-a@dent.tohoku.ac.jp (A.Y.); fukumoto@dent.tohoku.ac.jp (S.F.); 2 Section of Oral Medicine for Children, Division of Oral Health, Growth and Development, Faculty of Dental Science, Kyushu University, Fukuoka 812-8582, Japan

**Keywords:** hypersensitivity, tooth sensitivity, desensitizing agent, 4-methacryloxyethyl trimellitic acid (4-MET), calcium salt of 4-methacryloxyethyl trimellitic acid (C-MET), 10-methacryloyloxydecyl dihydrogen calcium phosphate (MDCP), randomized controlled trial (RCT)

## Abstract

Recently, the development of dental materials has increased the availability of various hyperesthesia desensitizers. However, there are no studies on the duration of retreatment in terms of adherence rates. Thus, the adhesion rates of resin-based desensitizers were investigated. We used a conventional desensitizer and a recently developed desensitizer containing calcium salt of 4-methacryloxyethyl trimellitic acid (C-MET) and 10-methacryloyloxydecyl dihydrogen calcium phosphate (MDCP). These colored agents were applied to the surfaces of premolars and molars, and the area was measured from weekly oral photographs. Areas were statistically analyzed and mean values were calculated using 95% confidence intervals. A *p*-value of <0.05 was considered statistically significant. These rates were significantly higher on the buccal side of the maxilla and lower on the lingual side of the maxilla. In addition, the desensitizer containing C-MET and MDCP displayed significantly higher adhesion rates. It is suggested that this will require monthly follow-ups and reevaluation because both agents cause less than 10% adherence and there is almost no sealing effect after 4 weeks. In addition, the significantly higher adhesion rate of the desensitizer containing C-MET and MDCP indicated that the novel monomer contributed to the improvement in the adhesion ability.

## 1. Introduction

Amelogenesis imperfecta (AI) is a congenital disorder characterized by an abnormal enamel formation on the crown of teeth. Researchers have identified mutations in genes, such as amelogenin, ameloblastin, matrix metalloproteinase 20, and kallikrein-related peptidase 4, as causes of AI [1,2,3,4]. AI is classified into four types according to their clinical appearances. Type 1 Hypoplastic is characterized by hard enamel; however, it is thin and may have pits. In contrast, AI of hypomaturation (type 2), hypocalcified (type 3), and hypomature hypoplastic enamel with taurodontism (type 4) manifests extremely brittle and fast-to-wear enamel, concomitant with pain. Genetic factors affect all permanent teeth, not only primary teeth. Furthermore, permanent molar incisor hypomineralization (MIH), hypomineralized second primary molars (HSPM), and hypoplasia-associated severe early childhood caries are not genetic but the systemic factors of abnormal enamel formation [5,6,7]. Ameloblasts differentiate through the secretory, mineralization, and maturation stages for enamel formation. Enamel hypomineralization occasionally develops because of a failure between the mineralization and maturation stages. Therefore, it may be attributed to effects during maternal pregnancy, the perinatal period, and the first year following birth. Discolored patches of soft enamel with hypersensitivity in these diseases occasionally appear symmetrically on the left and right or on the upper and lower sides. In contrast to genetic hypomineralization, such as AI, there is no genetic background, but complications during pregnancy such as diabetes and hypertension, vitamin D deficiency due to decreased sun exposure, nutrition problems such as malnutrition and obesity, tobacco and alcohol consumption, neonatal complications, low birth weight, and medications during infancy may also have an effect [8,9,10,11]. Some MIH can be painful owing to severe enamel defects [12,13]. The calcification period of second primary molars, permanent incisors, and first permanent molars is similar. Thus, 20% of HSPM will result in MIH, so dentists should be cautious with future permanent teeth in cases of primary tooth hypomineralization, owing to systemic factors [14]. By contrast, local calcification defects in permanent teeth are induced by mechanical stresses, namely trauma or caries in the primary teeth [15,16,17,18]. Hypomineralization appears as asymmetric discolored patches of soft or decayed sensitive bumpy enamel, and causes pain [19]. Moreover, enamel hypoplasia displays an increasing incidence [20,21]; therefore, dentists will have greater opportunities to perform hypersensitivity treatments on pediatric patients.

Hypersensitivity is also observed in adults. This is because it is not only induced by hypomineralization but also by abrasion, enamel attrition, tooth chips, cracked teeth, wedge-shape deficits (WSD), and the exposure of root dentin [22]. Most cracks caused by occlusal pressure are painful [23]. Generally, pain appears following dentin exposure, such as in WSD. Therefore, it is termed as dentin hypersensitivity. Contrarily, dental trauma, the removal of orthodontic brackets, whitening, and occlusal pressure cause enamel cracks [24,25,26,27]. The prognosis of cracked teeth with reversible pulpitis is relatively healthy [28,29]. Therefore, a reversible course of treatment is the first choice. Dentists may consider irreversible treatment with restorations if there is no improvement. Debonding with different orthodontic pliers substantially increases the number, length, and width of enamel micro cracks [25,30]. In this case, it is difficult to identify the micro crack area because there is no obvious dentin exposure. Tooth hypersensitivity is the most common side effect of tooth whitening [31]. In particular, it is not induced by office bleaching, but rather by home bleaching and frequent use [32,33]. Hydrogen peroxide and carbamide oxide are the primary agents of whitening. Whitening agents are characterized by a biochemical reaction that triggers the rupture of pigmented molecules impregnating the tooth structures, thereby lightening them. In other words, whitening agents infiltrated into the enamel are capable of producing morphologic changes to the structure or molecular composition of the tooth [34,35,36]. However, a report demonstrated that these agents did not affect the surface quality of enamel [37]. Therefore, the details of the mechanism underlying the induction of hyperesthesia by whitening agents are unclear; nonetheless, the mechanism is presumably different from dentin hypersensitivity [38]. However, fluoride and diode laser are also effective in whitening-induced hypersensitivity [39]. In this case, it is difficult to identify the site in the absence of obvious dentin exposure or caries, similar to orthodontic bracket removal. Therefore, dentifrices containing hypersensitivity desensitizers are applied to the entire tooth surface. Dentists consider the hydrodynamic theory following the exposure of an open dentin tube to an external stimulus, pressure changes in the dentin, and changes in the fluid flow in the tube, thereby resulting in nerve stimulation. Therefore, the majority of therapeutic agents are developed to seal open dentin tubules [40]. Thus, dentists apply hypersensitivity-inhibiting treatments with low-power diode lasers, sodium fluoride varnish, and occluding dentinal tubule agents [41,42,43]. They use oxalic acid, glutaraldehyde, and resin systems as the sealing agents of dentin tubules. Oxalic acid reacts with dental hydroxyapatite to form insoluble calcium oxalate crystals. By contrast, glutaraldehyde coagulates proteins, whereas resin physically seals the dentin tubules. Infrared diode laser, fluoride, and resin-based desensitizers are effective for not only dentin hypersensitivity but also enamel hypomineralization [44,45]. Sealing desensitizes the pulp, which has become sensitive with a lowered threshold. The desensitizing effect of hyperesthesia immediately disappears following application; however, it tends to increase with time [45,46,47]. Thus, the duration of suppression is unclear, and the re-treatment period is vague [47,48,49,50]. Recently, researchers have developed desensitizing agents for hypersensitivity containing Bioactive Monomer^TM^. The Bioactive Monomer^TM^ consists of a calcium salt of 4-methacryloxyethyl trimellitic acid (C-MET) and 10-methacryloyloxydecyl dihydrogen calcium phosphate (MDCP). The 4-MET binds to the calcium in the hydroxyapatite of teeth [51]. With the addition of 10% or less of C-MET, the calcium salt of 4-MET, formed a 5–20 micron resin tag in the dentinal tubules and did not reduce tensile strength, while hydroxyapatite was induced on the surface [52]. Despite having thin and tight adhesion in vitro [42], the adhesion potential of this novel agent is unknown in vivo. Therefore, the adhesion rate in vivo was evaluated using a new method in which colored resin-based desensitizing agents were prepared, applied to the tooth surface, and the colored area was measured from a digital photograph.

## 2. Materials and Methods

### 2.1. Ethical Aspects

Hybrid Coat II (HC) (70926000) (Sun Medical, Shiga, Japan) and Bio Coat Ca (BC) (70926000) (Sun Medical, Shiga, Japan) are approved in Japan as hypersensitivity inhibitors. The Ethical committee of Tohoku University (2020-3-004) approved the study protocol. Written informed consent that described the purpose and scope of this study was provided by all participants. The study design was a double blind, split-mouth, randomized controlled trial. It was registered at the University Hospital Medical Information Network (UMIN000044757).

### 2.2. Participant Selection

The sample was composed of the pre-molars and molars of 13 people (192 teeth) who were visiting the Tohoku University Dental Hospital. We applied two hypersensitivity inhibitor materials to the tooth surface in a split-mouth setting to participants aged 27 to 47 years. Those with physical or mental disabilities were excluded. We excluded caries according to the international caries detection and assessment system criteria in participants with a caries score ≥3.

### 2.3. Experimental Materials

HC is a single-bottle light-curing coating material. It is applied for surface coating of tooth surfaces with hypersensitivity. It comprises 4-methacryloxyethyl trimellitate anhydride (4-META), a high performance adhesive monomer that decalcifies the tooth substrate and penetrates through the smear layer to form a hybrid layer. BC is a Hybrid Coat with Bioactive Monomer^TM^, which includes a calcium salt of C-MET and MDCP (Table 1). Considering the transparency of these agents, we could not detect adhesion. Therefore, we added chromophthal red, a colorant used in common dental materials.

### 2.4. Allocation

In each unit, we used random numbers to decide the left or right side and HC or BC. The participants were blinded to each treatment during the study. Dentist A maintained a randomized allocation table, which was blinded to dentists B, C, D, and E.

### 2.5. Treatment Protocol

The tooth surfaces were mechanically cleaned using a brush and non-fluoride toothpaste for 10 min. Subsequently, the teeth were rinsed with water for approximately 1 min. Excess moisture was removed from the tooth surface using cotton pellets and air brow. One liquid drop was chemically reacted with a specific brush by mixing it for 5 s. The tooth surface was coated and maintained for 10 s. Air was blown for 5 s to thinly spread the liquid, and consequently cured by light irradiation. The light was irradiated for 20 s at 1200 mw/cm^2^ by G-Light Prima II Plus (GC, Tokyo, Japan), an LED irradiator with 2 wavelengths of 465 nm and 400 nm. The non-polymerized layer on the tooth surface was wiped with an alcohol cotton ball. The steps were performed by dentist B, with expertise in dental treatment. Both reagents had the identical protocol and color; thus, the operator was blinded.

### 2.6. Evaluation

**Figure 1:**
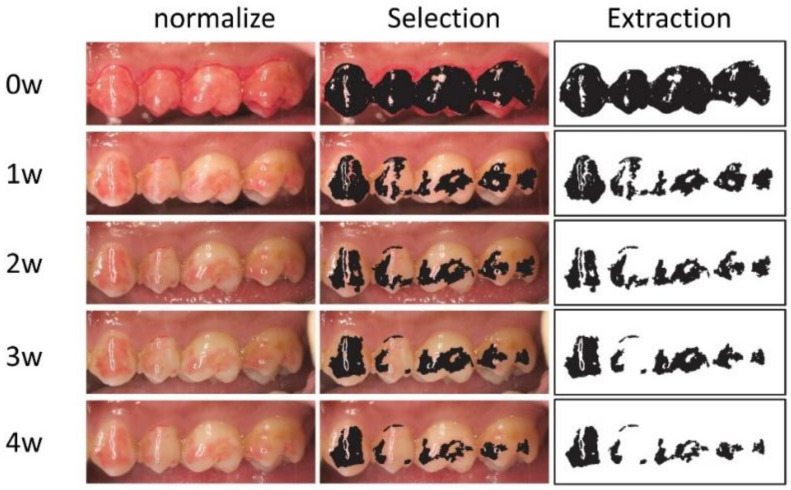
Intraoral photograph forms and adjusted color tones.

Dentist C captured intraoral photographs weekly, and followed up with them for 4 weeks. The photographer was blinded to the process. All teeth were followed up, and there were no dropouts. All intraoral images were captured using a digital camera according to the commonly adopted method [53]. We objectively evaluated the adhesion ability by previously described methods [45]. Each photograph was normalized for the color tone, white balance, contrast, size, and angle. Moreover, each red-colored region was extracted using Photoshop CS6 (Adobe, San Jose, CA, USA) by dentist D. The extracted areas were measured in triplicate by ImageJ (http://imagej.nih.gov/ij/, accessed on 11 November 2015) (Figure 1). The measured values were allocated by dentist A to the HC and BC groups.

### 2.7. Statistical Analysis

Data were entered into a database and analyzed using the GraphPad Prism9 (MDF Co., Ltd., Tokyo, Japan) by dentist E. Dentist E was not informed of each group and was blind. The HC and BC groups underwent a retention assessment of the resin-based desensitizing agents. Moreover, each group was analyzed by classifying the oral region into eight areas (maxillary, mandible, lingual, buccal, premolar, and molar). A *p*-value < 0.05 at 95% confidence interval (CI) was considered as statistically significant.

## 3. Results

### 3.1. The Time Course of Adhesion Rate of Two Types of Resin-Based Desensitizing Agents by Their Site

#### 3.1.1. Adhesion Ability of Hybrid Coat II

The adhesion rate in the HC group decreased to 12.24% (95%CI 13.71–10.77%) and 3.11% (95%CI 3.72–2.49%) at 1 week and 4 weeks, respectively (Table 2). We observed significantly higher adhesion rates for all periods on the buccal side of the maxillary premolars and maxillary molars in eight areas (Table 2). Thus, the maxillary buccal attachment rate increased (Table 3). However, there was no significant difference between the maxilla and mandible. In addition, the adhesion rate was higher on the buccal side in some periods (Table 3). By contrast, we observed a significant decrease in the adhesion rate on the buccal side of mandibular molars, the lingual side of maxillary premolars, and the lingual side of maxillary molars in some periods (Table 2). Therefore, the rate of adhesion decreased in the lingual surface of the maxilla and lingual surfaces for all periods, and in the mandibular for some periods (Table 3). Among the 8 regions, mandibular lingual premolars, mandibular lingual molars, and mandibular buccal premolars had similar whole attachment rates. There was no significant difference between the premolars and molars. We captured an occlusal surface photograph using a mirror; however, it was almost completely removed within a week (data not shown).

#### 3.1.2. The Adhesion Ability of Bio Coat Ca

The adhesion rates in the BC group were 28.31% (95%CI 31.24–25.38%) and 9.03% (95%CI 10.55–7.51%) at 1 week and 4 weeks, respectively. In all periods, the rates were significantly higher on the buccal surface of the maxillary premolars and maxillary molars in eight areas (Table 4). Thus, adhesion rates were high at the buccal side of the maxilla, maxilla, and buccal side in all periods (Table 5). In contrast, we observed significantly reduced adhesion on the buccal surface of the mandibular molars for all periods, and on the lingual surface of the mandibular premolars and molars for some periods (Table 4). The rates were significantly lower in the lingual and buccal sides of the mandible in four areas; thus, the adhesion rates were low in the mandibular and lingual sides in the two divided regions (Table 5). Among the 8 regions, mandibular buccal premolars, maxillary lingual premolars, and maxillary lingual molars were similar to the whole attachment rate. We observed no significant difference between the premolars and molars in the BC group (Table 5). The majority of occlusal surfaces detached within a week (data not shown). The rate of adhesion by the site was similar between the HC and BC groups; nonetheless, the result was more apparent in the BC group.

### 3.2. Differences in the Adhesion Ability of the Resin-Based Desensitizing Agents

We compared the adhesion rates in HC (Table 2) and BC (Table 4), and determined significant differences (Table 6). There was no significance on the occlusal surface as both agents were almost exfoliated in 1 week. The lingual side of the mandibular premolar and the buccal side of the mandibular molar were not significantly different between HC and BC in all periods. Nonetheless, the lingual surface of the mandibular molar was marginally different. However, the adhesion of the BC group was considerably higher at other sites, in addition to a remarkable difference between the buccal side of the maxillary premolar and molar. We compared the findings from Table 3 and Table 5, and calculated significant differences (Table 7). The adhesion rate of the BC group was higher in the lingual and buccal sides of the mandible, whereas it was more pronounced in the lingual and buccal sides of the maxilla. In addition, the BC group demonstrated a substantially increased adhesion rate in the maxillary, mandibular, buccal, lingual, premolar, molar, and whole teeth.

## 4. Discussion

A one-step bonding agent has been developed to simplify the operating procedure for repairing composite resin. These agents not only consist of acidic, hydrophilic, and multifunctional monomers with phosphate or carboxylic groups in the molecule, but also organic solvents and water. The acidic multifunctional monomers etch hydroxyapatite and a carboxylic monomer penetrates into the tooth surface to form a hybrid layer of dentin or an enamel resin tag to enhance adhesion to the resin. The method of use involves applying the self-etching primer to the teeth, leaving it on for 20 s to 30 s, and drying without rinsing, which is different from the usual acid treatment. However, the two-step process with etching and rinsing is more adhesive [54], so the target is to increase the adhesive strength of the one-step bond. The phosphoric acid monomer, 10-methacryloyloxydecyl dihydrogen phosphate (MDP), demineralizes dentin apatite, whereas the hydrophilic monomer, 2-hydroxyethylmethacrylate (HEMA), primes the exposed dentin collagen fibers. By contrast, multifunctional monomers, urethane dimethacrylate (UDMA) and triethylene glycol dimethacrylate (TEGDMA), improve the mechanical strength of bonding by resin-cured materials. The majority of monomers used in the adhesive system and composite resin, such as TEGDMA and HEMA, demonstrate cytotoxic effects on odontoblasts and fibroblasts in vitro [55,56]. In recent years, 4-MET, which is a monomer that contains carboxylic functional groups, has displayed dental adhesive properties and is currently used in numerous dental materials. It is not only adhesive but also contains additional multifunctional monomers with durable and ion-sustained release properties [57,58]. Researchers reported on a split-mouth randomized, double-blind clinical trial comparing fluoride varnish and bonding resin for hypersensitivity [59]. Dentin hypersensitivity was improved in both groups. However, resin demonstrated effective pain improvement and long-term impact. Therefore, the resin system should have a high potential as a hyperesthesia inhibitor. HC containing 4-META is an effective agent that suppresses hyperesthesia [60]. Being the core component of HC, 4-META could promote the penetration of monomers into a demineralized tooth structure. Recently, BC has been developed as a novel self-etch adhesive resin material that inhibits hypersensitivity. CMET is a calcium salt of 4-MET and is used as an adjunctive component in a commercialized adhesive system. It exhibits high adhesive strength, inhibits biofilm formation, and allows remineralization, besides displaying low cytotoxicity in vitro [42,61,62,63]. A previous in vitro study which tested micro tensile bonds with dentin at 24 h, 3 months, 6 months, and 2 years, yielded results of 41.3, 34.0, 27.9, and 22.6 MPa in the C-MET group compared with 30.9, 28.7, 19.8, and 13.5 MP in the 4-META group, indicating that the bond strength of the CMET group was higher over all periods [64]. A previous clinical study reported on substantially reduced pain immediately following BC application for hypersensitivity using the visual analog scale (VAS) for pain evaluation [45]. However, minor pain recurred 1 month later, and the patient was retreated. Following monthly application, VAS decreased for the first 4 months but did not change thereafter. Repeated applications were effective in severe hyperesthesia; nevertheless, the interval between the applications remained unclear. Therefore, we evaluated the adherence rate of novel agents in this randomized controlled clinical trial using a split-mouse approach. In both groups, more than half and <10% of the area detached in 1 week and 4 weeks, respectively. The attrition rate was higher in the mandible than that in the maxilla, and in the lingual than that in the buccal region. However, there was no difference between the molars and premolars. The higher rate of detachment on the lingual surface is presumably caused by an abrasion by the tongue. In addition, occlusal pressure may affect the lingual surface of the maxilla. Likewise, the buccal surface of the mandible also displayed higher detachment rates. Contrarily, the avulsion rate was low on the buccal side of the maxilla. The buccal side of the maxilla may be less affected by mucosal scraping. WSD is observed in the premolar and buccal regions, and rarely in the lingual region of the mandible [65,66]. The occlusal pressure did not induce cracks on the lingual side [67]. Moreover, orthodontic brackets are commonly attached to the labial side. By contrast, the crack may be located on the lingual side in the case of lingual brackets or trauma. Taken together, dentists may consider the interval between clinic visits depending on the site of application. The percentage of occlusal attachment decreased under the detection limit after 1 week, so it may be less effective in reducing pain caused by attrition. This can be attributed to differences in the attrition rate according to the site. BC containing the novel Bioactive Monomer^TM^ exhibited significantly higher adherence rates compared with HC. The neutralization of the strong acidity of 4-MET by the replacement of the hydrogen ions on the two carboxyl groups may reduce the etching effect. However, it exhibited a high adherence rate in vitro. There were no significant differences on the lingual side of the mandibular premolars; nonetheless, the overall C-MET displayed a higher rate of attachment. Thus, we considered C-MET as a high-performance monomer that not only improves retention in vitro but also clinically [52,64]. 

Self-etching primers containing 4-META penetrate dentin tubules to form strong resin tags, resulting in better adhesion to dentin than enamel [68,69]. Generally, the adhesion of 4-MET on the enamel is more effective when etched with tooth surface by phosphoric acid. We did not perform phosphate etching to avoid excessive invasiveness. Therefore, if malformation or crack location is clearly identified, the use of partial phosphoric acid should increase the retention effect on the enamel. Furthermore, the dentin gets exposed in the case of WSD; thus, the adhesion rate and effect of desensitization are likely to be higher.

In a traditional in vitro study, enamel and dentin discs are carved from the molars and coated with an agent. The sample is fixed to the jig with cyanoacrylate adhesive and a tensile load is applied. Micro-tensile bond strength (Unit: MPa) is calculated from the maximum load and the area of the cross section. The surface texture of the cross section is evaluated by scanning electron microscopes (SEM). On the other hand, it is difficult to reproduce the oral environment in vitro. In this study, we developed a new method to apply a colored desensitizer to the tooth surface in vivo. We calculated the area of adhesion from intraoral photographs, which may not be an exact value owing to limitations in the normalization and extraction of photographs. However, we identified significant differences in diverse areas and agents. The aforementioned method of measurement may be useful as a brief test. Among the eight regions, the buccal surfaces of the mandibular premolars in both the BC and HC groups are similar to the whole tooth surface with no correlation, so this would be a suitable region to evaluate the attachment rate of individuals. We did not provide any intervention with food or brushing. The participants had three meals a day and brushed thrice daily with a hand toothbrush with toothpaste. Food, manual toothbrushes, and toothpaste types were not restricted. Because of the small scale of this study, the adherence rates were unrelated to mealtime and brushing time. Larger studies may lead to more correlations and evidence. This is an RCT study measuring the rate of adhesion of resin-based desensitizers for hypersensitivity, and the correlation with pain suppression is unknown. This new bioactive monomer forms hydroxyapatite on the tooth surface [42], which may provide more sustained pain suppression than 4-META. In the future, treatment intervals will be determined by applying desensitizers to hypersensitive teeth and measuring the adhesion rate and pain suppression effect using the VAS for each tooth region.

## 5. Conclusions

1. The adhesion rate of both resin-based desensitizing agents became 10% or less after one month.
2. Adhesion rates decreased on the mandibular and lingual sides, especially on the mandibular lingual side.
3. Adhesion rates were higher in the maxillary and buccal regions, especially in the maxillary buccal region.
4. Addition of C-MET and MDCP contributed to improvements of adhesion rate.

## Figures and Tables

**Table 1:** Composition of Hybrid Coat II (HC) and Bio Coat Ca (BC).

HC	Liquid	acetone, methacrylic monomers (methyl methacrylate, 4-META), water, initiator
	Brush	aromatic amine, aromatic sulfinate salt
BC	Liquid	acetone, methacrylic esters (4-META), water, initiator
	Brush	aromatic amine, aromatic sulfinate salt, C-MET, MDCP

**Table 2:** The average Hybrid Coat adhesion rate (%) of the area divided into eight sections.

	Mand	Mand	Mand	Mand	Maxi	Maxi	Maxi	Maxi	
	Pre	Mol	Pre	Mol	Pre	Mol	Pre	Mol	Whole
	Ling	Ling	Bucc	Bucc	Ling	Ling	Bucc	Bucc	
	(*n* = 24)	(*n* = 26)	(*n* = 24)	(*n* = 26)	(*n* = 20)	(*n* = 26)	(*n* = 20)	(*n* = 26)	(*n* = 192)
1 week	12.25	10.14	13.66	5.35 *	6.50 *	7.83	21.94 *	20.84 *	12.24
(95%CI)	(15.99–8.50)	(13.08–7.21)	(17.38–9.95)	(6.99–3.70)	(9.63–3.37)	(11.22–4.44)	(26.61–17.28)	(24.76–16.93)	(13.71–10.77)
2 weeks	5.83	5.77	8.43	3.37 *	3.38	3.30 *	18.10 *	13.81 *	7.57
(95%CI)	(7.69–3.07)	(8.16–3.37)	(10.95–5.91)	(5.02–1.72)	(5.64–1.13)	(5.11–1.48)	(22.20–14.00)	(17.49–10.12)	(8.75–6.39)
3 weeks	3.04	3.01	4.28	2.07	2.67	1.18 *	11.82 *	8.66 *	4.57
(95%CI)	(4.43–1.65)	(4.19–1.84)	(5.38–3.17)	(3.17–0.98)	(4.32–1.01)	(2.77–0.86)	(15.10–8.55)	(10.78–6.55)	(5.32–3.82)
4 weeks	2.29	2.13	2.28	1.35	1.51	1.15 *	8.50 *	6.22 *	3.11
(95%CI)	(3.47–1.11)	(3.05–1.20)	(3.24–1.32)	(2.24–0.47)	(2.64–0.37)	(1.83–0.48)	(11.40–5.60)	(8.30–4.15)	(3.72–2.49)

The regions were divided into maxillary (Maxi) and mandible (Mand), premolar (Pre) and molar (Mol), and lingual (Ling) and buccal (Bucc) sides. * p < 0.05.

**Table 3:** The average Hybrid Coat adhesion rate (%) by the region.

	Mand	Mand	Maxi	Maxi	Mand	Maxi	Ling	Bucc	Pre	Mol
	Ling	Bucc	Ling	Bucc						
	(*n* = 50)	(*n* = 50)	(*n* = 46)	(*n* = 46)	(*n* = 100)	(*n* = 92)	(*n* = 96)	(*n* = 96)	(*n* = 88)	(*n* = 104)
1 week	11.20	9.51	7.22 *	21.35 *	10.35	14.29	9.29 *	15.19	13.54	11.04
(95%CI)	(13.58–8.81)	(11.82–7.19)	(9.57–4.88)	(24.36–18.34)	(12.02–8.68)	(16.68–11.89)	(11.01–7.57)	(17.41–12.97)	(15.75–11.32)	(12.96–9.13)
2 weeks	5.57	5.90	3.34 *	15.77 *	5.74	9.56	4.50 *	10.64 *	8.66	6.56
(95%CI)	(7.24–3.91)	(7.55–4.25)	(4.76–1.91)	(18.58–12.96)	(6.91–4.56)	(11.58–7.53)	(5.63–3.37)	(12.52–8.76)	(10.48–6.85)	(8.06–5.05)
3 weeks	3.03	3.18	2.20 *	10.11 *	3.10 *	6.16	2.63 *	6.50 *	5.30	3.89
(95%CI)	(3.93–2.12)	(4.01–2.34)	(3.12–1.29)	(12.04–8.18)	(3.72–2.48)	(7.50–4.82)	(3.28–1.98)	(7.74–5.27)	(6.53–4.08)	(4.78–3.00)
4 weeks	2.21	1.82	1.32 *	7.27 *	2.01 *	4.29	1.78 *	4.43	3.53	2.71
(95%CI)	(2.96–1.46)	(2.48–1.15)	(1.95–0.69)	(9.03–5.51)	(2.52–1.51)	(5.41–3.18)	(2.28–1.28)	(5.50–3.37)	(4.53–2.53)	(3.46–1.97)

The premolars and molars are integrated and divided into four regions, specifically into two separate sections, such as maxillary (Maxi) and mandible (Mand), buccal (Bucc) and lingual (Ling), and molar (Mol) and premolar (Pre). * *p* < 0.05.

**Table 4:** The average Bio Coat Ca adhesion rate (%) of the area divided into eight sections.

	Mand	Mand	Mand	Mand	Maxi	Maxi	Maxi	Maxi	
	Pre	Mol	Pre	Mol	Pre	Mol	Pre	Mol	Whole
	Ling	Ling	Bucc	Bucc	Ling	Ling	Bucc	Bucc	
	(*n* = 24)	(*n* = 26)	(*n* = 24)	(*n* = 26)	(*n* = 20)	(*n* = 26)	(*n* = 20)	(*n* = 26)	(*n* = 192)
1 week	20.79	17.69 *	27.36	9.55 *	30.06	20.33	53.82 *	51.06 *	28.31
(95%CI)	(27.38–14.21)	(22.56–12.82)	(34.07–20.65)	(13.19–5.91)	(37.60–22.51)	(26.58–14.08)	(58.76–48.88)	(56.81–45.31)	(31.24–25.38)
2 weeks	9.28 *	7.94 *	15.88	5.50 *	19.50	15.03	42.14 *	38.30 *	18.73
(95%CI)	(12.52–6.04)	(10.85–5.03)	(20.58–11.18)	(9.44–1.56)	(26.66–12.35)	(21.23–8.83)	(49.78–34.51)	(43.06–33.54)	(21.29–16.18)
3 weeks	6.17 *	6.29 *	10.52	4.49 *	14.69	10.13	28.74 *	29.01 *	13.44
(95%CI)	(8.34–4.01)	(8.79–3.79)	(13.99–7.05)	(7.47–1.50)	(20.29–9.10)	(15.13–5.13)	(34.61–22.86)	(33.44–24.58)	(15.37–11.50)
4 weeks	4.13	4.58	5.24	2.12 *	8.22	4.98	22.35 *	22.55 *	9.03
(95%CI)	(5.88–2.37)	(6.35–2.81)	(6.95–3.53)	(3.65–0.59)	(11.06–5.39)	(7.04–2.91)	(28.43–16.27)	(26.99–18.12)	(10.55–7.51)

The regions were divided into maxillary (Maxi) and mandible (Mand), premolar (Pre) and molar (Mol), and lingual (Ling) and buccal (Bucc) sides. * *p* < 0.05.

**Table 5:** The average Bio Coat Ca adhesion rate (%) by the region.

	Mand	Mand	Maxi	Maxi	Mand	Maxi	Ling	Bucc	Pre	Mol
	Ling	Bucc	Ling	Bucc						
	(*n* = 50)	(*n* = 50)	(*n* = 46)	(*n* = 46)	(*n* = 100)	(*n* = 92)	(*n* = 96)	(*n* = 96)	(*n* = 88)	(*n* = 104)
1 week	19.24 *	18.46 *	24.79	52.33 *	18.85 *	38.56 *	21.90 *	34.71 *	32.26	24.66
(95%CI)	(23.34–15.15)	(22.97–13.95)	(29.81–19.76)	(56.22–48.43)	(21.90–15.80)	(42.80–34.31)	(25.17–18.64)	(39.24–30.19)	(36.42–28.10)	(28.66–20.66)
2 weeks	8.61 *	10.69 *	17.08	40.06 *	9.65 *	28.57 *	12.68 *	24.79 *	20.94	16.69
(95%CI)	(10.79–6.43)	(14.07–7.31)	(21.81–12.35)	(44.40–35.72)	(11.67–7.63)	(32.55–24.59)	(15.35–10.00)	(28.79–20.78)	(24.75–17.13)	(20.09–13.30)
3 weeks	6.23 *	7.50 *	12.22	28.88 *	6.87 *	20.55 *	9.11 *	17.77 *	14.47	12.48
(95%CI)	(7.89–4.57)	(9.93–5.07)	(16.01–8.44)	(32.48–25.29)	(8.34–5.39)	(23.67–17.44)	(11.20–7.01)	(20.79–14.74)	(17.25–11.70)	(15.17–9.78)
4 weeks	4.35 *	3.68 *	6.46	22.46 *	4.02 *	14.46 *	5.37 *	12.70 *	9.54	8.56
(95%CI)	(5.60–3.10)	(4.91–2.46)	(8.23–4.70)	(26.12–18.80)	(4.89–3.14)	(17.07–11.85)	(6.45–4.28)	(15.34–10.05)	(11.78–7.30)	(10.63–6.48)

The premolars and molars are integrated and divided into four regions, specifically into two separate sections, such as maxillary (Maxi) and mandible (Mand), buccal (Bucc) and lingual (Ling), and molar (Mol) and premolar (Pre). * *p* < 0.05.

**Table 6:** *p*-values comparing the adhesion ratio of Bio Coat Ca and Hybrid Coat II in eight sections.

	Mand	Mand	Mand	Mand	Maxi	Maxi	Maxi	Maxi	
	Pre	Mol	Pre	Mol	Pre	Mol	Pre	Mol	Whole
	Ling	Ling	Bucc	Bucc	Ling	Ling	Bucc	Bucc	
	(*n* = 24)	(*n* = 26)	(*n* = 24)	(*n* = 26)	(*n* = 20)	(*n* = 26)	(*n* = 20)	(*n* = 26)	(*n* = 192)
1 week	0.0655	0.0450 *	0.0104 *	0.0872	0.0004 *	0.0140 *	1.2 × 10^−6^ *	2.9 × 10^−6^ *	3.5 × 10^−11^ *
2 weeks	0.0935	0.2251	0.0323 *	0.2564	0.0037 *	0.0118 *	0.0050 *	7.3 × 10^−6^ *	3.9 × 10^−8^ *
3 weeks	0.0520	0.0638	0.0129 *	0.1615	0.0049 *	0.0198 *	0.0012 *	5.7 × 10^−6^ *	4.5 × 10^−9^ *
4 weeks	0.1208	0.0578	0.2356 *	0.2842	0.0032 *	0.0140 *	0.0050 *	8.7 × 10^−6^ *	4.4 × 10^−7^*

The regions were divided into maxillary (Maxi) and mandible (Mand), premolar (Pre) and molar (Mol), lingual (Ling) and buccal (Bucc) sides. * *p* < 0.05.

**Table 7:** *p*-values comparing the adhesion ratio of Bio Coat Ca and Hybrid Coat II in each area.

	Mand	Mand	Maxi	Maxi	Mand	Maxi	Ling	Bucc	Pre	Mol
	Ling	Bucc	Ling	Bucc						
	(*n* = 50)	(*n* = 50)	(*n* = 46)	(*n* = 46)	(*n* = 100)	(*n* = 92)	(*n* = 96)	(*n* = 96)	(*n* = 88)	(*n* = 104)
1 week	0.0113 *	0.0090 *	3.3 × 10^−5^ *	1.3 × 10^−11^ *	0.0005 *	2.1 × 10^−10^ *	3.0 × 10^−6^ *	2.1 × 10^−7^ *	7.2 × 10^−6^ *	2.7 × 10^−5^ *
2 weeks	0.0658	0.0417 *	0.0002 *	2.5 × 10^−8^ *	0.0105 *	2.1 × 10^−8^ *	7.5 × 10^−5^ *	1.0 × 10^−5^ *	3.5 × 10^−6^ *	0.0002 *
3 weeks	0.0114 *	0.0119 *	0.0004 *	3.8 × 10^−8^ *	0.0007 *	2.3 × 10^−8^ *	3.8 × 10^−5^ *	2.5 × 10^−6^ *	1.8 × 10^−6^ *	3.4 × 10^−5^ *
4 weeks	0.0233 *	0.0347 *	0.0002 *	2.4 × 10^−6^ *	0.0033 *	1.2 × 10^−6^ *	3.1 × 10^−5^ *	5.0 × 10^−5^ *	0.0003 *	0.0002 *

The premolars and molars are integrated and divided into four regions, which are classified into two separate sections, such as maxillary (Maxi) and mandible (Mand), buccal (Bucc) and lingual (Ling), molar (Mol) and premolar (Pre). * *p* < 0.05.
